# Integrating Safety Culture and Quality Improvement in Paediatric Emergency Departments: Evidence From a Systematic Review

**DOI:** 10.7759/cureus.96959

**Published:** 2025-11-16

**Authors:** Wiaam Noureldin Birima Noureldin, Hammam Noureldin, Enas Noureldin

**Affiliations:** 1 Paediatric Emergency, Bristol Royal Hospital for Children, Bristol, GBR; 2 Emergency Medicine, University Hospital Limerick, Limerick, IRL; 3 General Practice, Alzaiem Alazhari University, Khartoum, SDN

**Keywords:** healthcare quality, patient safety, pediatric emergency department, quality improvement, safety culture, systematic review

## Abstract

Patient safety and quality improvement (QI) are critical pillars in paediatric emergency departments (PEDs), where high-stakes care increases vulnerability to errors. While fostering a strong safety culture is recognised as essential, its practical integration with QI initiatives is not fully understood. This systematic review aimed to synthesise evidence on how safety culture principles and QI strategies are integrated within PEDs and to assess their impact on patient safety and care quality. This review was conducted following the PRISMA 2020 guidelines. A comprehensive search of PubMed, Scopus, Web of Science, Embase, and CINAHL was performed for studies published between 2020 and 2024. Eligible studies described initiatives integrating safety culture and QI in PEDs. Data on study characteristics, interventions, outcomes, and implementation factors were extracted. The methodological quality of included studies was assessed using the Quality Improvement Minimum Quality Criteria Set (QI-MQCS). Fourteen studies were included. The integration was primarily achieved through the standardisation of clinical processes, education and training, technological integration, system-level process redesign, and structured team communication. These initiatives consistently improved outcomes, including reduced unnecessary interventions, decreased procedure times and length of stay, lower blood culture contamination rates, and enhanced patient flow. Key facilitators were iterative QI methods like plan-do-study-act (PDSA) cycles, stakeholder engagement, and clinical champions, while common barriers included workflow resistance and challenges in maintaining adherence. Quality assessment revealed strong reporting on foundational elements but a notable lack of evidence regarding long-term sustainability and the spread of interventions to other settings. The integration of safety culture principles is a powerful enabler of successful quality improvement in the PED. Effective interventions are those that address socio-cultural elements like teamwork, staff engagement, and continuous learning alongside technical process changes. Future work must prioritise demonstrating the long-term sustainability and broader disseminability of these integrated approaches to foster a pervasive culture of safety and excellence in paediatric emergency care.

## Introduction and background

Patient safety and quality improvement (QI) have emerged as fundamental pillars in healthcare systems worldwide, particularly within paediatric emergency departments (PEDs), where the complexity and urgency of care can heighten the risk of medical errors [1]. Paediatric patients present unique challenges due to age-specific physiological differences, communication barriers, and reliance on caregivers, making the emergency setting inherently vulnerable to adverse events [2]. Therefore, fostering a strong safety culture, defined as the shared values, attitudes, and behaviours that prioritise safety, has become an essential strategy to enhance patient outcomes and optimise care delivery in paediatric emergency settings [3].

Safety culture encompasses various dimensions, including teamwork, communication openness, leadership support, and error reporting, all of which are instrumental in shaping how healthcare professionals perceive and respond to risks [4]. In PEDs, where rapid [5] and interdisciplinary coordination are critical, a positive safety culture not only mitigates harm but also supports continuous learning and system resilience. However, the translation of safety culture principles into practical and sustainable quality improvement initiatives remains a significant challenge for many institutions.

Quality improvement frameworks, such as plan-do-study-act (PDSA) cycles, lean methodologies, and Six Sigma, have been increasingly adopted in paediatric emergency care to streamline workflows, reduce variability, and improve safety outcomes [6]. When effectively integrated with safety culture interventions, these approaches can create a synergistic effect, empowering frontline staff, enhancing accountability, and promoting data-driven improvements [7]. Despite this potential, the degree and mechanisms through which safety culture integration influences the success of QI initiatives in PEDs remain inconsistently reported across the literature.

Recent studies have highlighted that organisational leadership, staff engagement, and measurement tools such as the Safety Attitudes Questionnaire (SAQ) or Hospital Survey on Patient Safety Culture (HSOPSC) play pivotal roles in determining the effectiveness of such integrations [8]. Yet, variations in study designs, outcomes, and contextual factors make it difficult to generalise findings or establish best practices. A comprehensive synthesis of available evidence is therefore essential to understand how safety culture and QI strategies intersect to improve patient safety in paediatric emergency care.

This systematic review aims to critically evaluate and synthesise the existing literature on the integration of safety culture and quality improvement initiatives within paediatric emergency departments. Specifically, it seeks to identify common approaches, measurement tools, barriers, and facilitators associated with implementation and to assess their impact on patient safety outcomes. Beyond summarising current evidence, this review is expected to generate actionable insights that can inform evidence-based strategies to strengthen organisational culture, enhance team communication, and reduce preventable adverse events in paediatric emergency settings. The findings will be particularly valuable for healthcare leaders, clinicians, and policymakers seeking to translate safety culture principles into measurable quality improvement practices, ultimately contributing to safer, more reliable, and child-centred emergency care.

## Review

Methodology 

Study Design and Protocol

This systematic review was conducted following the Preferred Reporting Items for Systematic Reviews and Meta-Analyses (PRISMA 2020) guidelines to ensure transparency and methodological rigour [9]. The review protocol was developed a priori to outline the search strategy, inclusion criteria, data extraction process, and quality assessment approach. The primary objective was to synthesise existing evidence on the integration of safety culture and QI strategies in PEDs, focusing on their implementation, measurement tools, barriers, facilitators, and outcomes related to patient safety and care quality.

Eligibility Criteria (PICOS Framework)

The eligibility criteria for study selection were defined according to the PICOS framework (population, intervention, comparison, outcomes, and study design) (Table 1).

**Table 1:** Eligibility criteria for studies selection as per the PICOS framework This table outlines the predefined inclusion criteria for the systematic review, structured according to the PICOS (population, intervention, comparison, outcomes, study design) framework.

PICOS Element	Description
Population (P)	Pediatric patients (aged 0–18 years) receiving care in hospital-based or standalone pediatric emergency departments.
Intervention (I)	Interventions or initiatives integrating safety culture and QI strategies aimed at improving patient safety, communication, teamwork, or clinical outcomes.
Comparison (C)	Studies comparing pre- and post-intervention outcomes, or between departments/units with varying levels of safety culture or QI implementation.
Outcomes (O)	Outcomes related to safety culture improvements, error reduction, staff perceptions, patient outcomes, or QI process effectiveness.
Study Design (S)	Quantitative, qualitative, and mixed-methods studies published in peer-reviewed journals between January 2020 and October 2025 to ensure inclusion of the most recent and relevant evidence. Reviews, editorials, and conference abstracts were excluded.

Information Sources and Search Strategy

A comprehensive literature search was conducted across four major databases: PubMed, Scopus, Web of Science, and Embase. These databases were selected for their broad coverage of biomedical and healthcare research. The search strategy combined Medical Subject Headings (MeSH) and free-text terms using Boolean operators (AND, OR) to capture studies related to “safety culture”, “quality improvement”, and “paediatric emergency department.” Search strings were tailored for each database to optimise sensitivity and specificity. The search was limited to studies published in English between 2020 and 2025 to capture contemporary developments and implementation trends in safety and quality improvement practices. Reference lists of all included studies were manually screened to identify additional relevant publications. The detailed search strategy for each database is provided in the Appendix, Table 5.

Study Selection

All retrieved references were imported into EndNote X9 (Clarivate Analytics) for efficient management and removal of duplicates. Following de-duplication, two independent reviewers screened the titles and abstracts based on the predefined eligibility criteria. Full-text articles were subsequently assessed for final inclusion. Disagreements between reviewers were resolved through discussion or by consultation with a third reviewer to ensure unbiased selection.

Data Extraction

A standardised data extraction form was developed to ensure consistency in data collection. Extracted information included author details, year of publication, country/setting, study design, sample characteristics, intervention type, safety culture dimension, quality improvement strategy, outcome measures, and key findings. Additional information regarding barriers, facilitators, and implementation frameworks was also captured where available. The extracted data were cross-verified by a second reviewer to ensure accuracy and completeness.

Risk of Bias and Quality Assessment

The methodological quality and risk of bias of included studies were assessed using the quality improvement minimum quality criteria set (QI-MQCS) tool [10]. This validated instrument evaluates QI-focused studies across 16 domains, including intervention description, study design, data source, outcomes, and sustainability. Each study was independently appraised by two reviewers, with disagreements resolved through consensus. Studies were categorised as high, moderate, or low quality based on their cumulative QI-MQCS scores.

Data Synthesis

Given the expected methodological heterogeneity among studies, in terms of study design, intervention type, outcome measures, and assessment tools, a meta-analysis was not performed. Instead, a narrative synthesis approach was used to qualitatively integrate findings across studies. This approach allowed for the exploration of patterns, themes, and contextual factors influencing the integration of safety culture and QI interventions in paediatric emergency settings. The decision to forego meta-analysis was justified by the diverse nature of the included studies, which precluded statistical pooling without compromising validity or interpretability.

Ethical Considerations

As this study synthesised data from previously published research, ethical approval was not required. However, all included studies were assumed to have obtained ethical clearance from their respective institutional review boards.

Results

Study Selection Process

**Figure 1:**
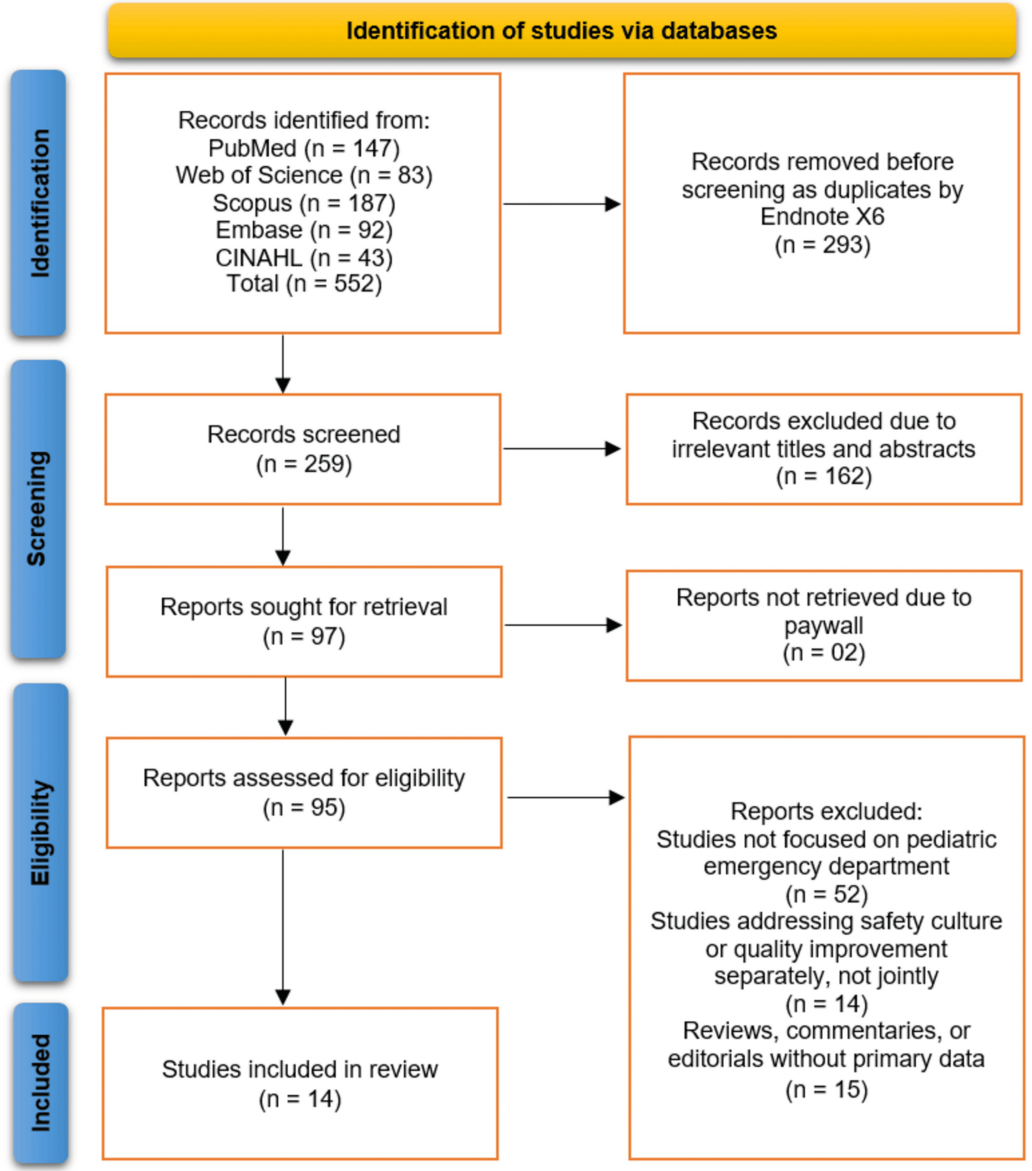
Studies selection process illustrated on PRISMA flowchart This diagram illustrates the systematic process of study identification, screening, eligibility assessment, and inclusion for the systematic review, detailing the number of records sourced from each database and the reasons for exclusion at each stage.

The study selection process is detailed in the PRISMA flowchart (Figure 1). A total of 552 records were identified through initial searches of electronic databases (PubMed: n=147, Web of Science: n=83, Scopus: n=187, Embase: n=92, CINAHL: n=43). After the removal of 293 duplicate records, 259 unique records were screened based on their titles and abstracts. This screening led to the exclusion of 162 records that were deemed irrelevant to the review's focus. The full text of the remaining 97 reports was sought for retrieval, of which 95 were successfully accessed; two reports could not be retrieved due to paywall restrictions. A detailed eligibility assessment of these 95 full-text articles was performed, resulting in the exclusion of 81 studies. The reasons for exclusion were as follows: 52 studies were not focused on the paediatric emergency department setting, 14 studies addressed either safety culture or quality improvement in isolation but not their integration, and 15 reviewed articles, commentaries, or editorials that did not present primary data. Ultimately, 14 studies met all inclusion criteria and were incorporated into the systematic review [11-24] (Figure 1).

Characteristics of Included Studies

The systematic review included 14 studies [11-24] that investigated the integration of safety culture principles with QI methodologies in PEDs. The characteristics of these studies are detailed in Table 2. The included studies were published between 2020 and 2024 and were conducted in a range of countries, including the United States, Canada, Turkey, and Italy. All studies employed QI frameworks, with the PDSA cycle being the most frequently utilised methodology [12,13,18,19,24]. Other approaches included lean methodology [22], the model for improvement [20], and simulation modelling [16]. The interventions targeted a diverse array of clinical processes and safety concerns, such as the management of specific conditions (e.g., croup [11], intussusception [18], febrile infants [20]), procedural efficiency (e.g., time to ultrasound [12], procedural sedation [14]), and system-level challenges (e.g., patient flow [22], physical restraint use [15], vaccination rates [24]). Sample sizes varied considerably, ranging from focused studies with under 100 encounters [18,20] to large-scale initiatives encompassing over 70,000 patient visits [22,24].

**Table 2:** Characteristics of included studies This table provides a comprehensive summary of the 14 studies included in the systematic review, detailing authors, setting, design, sample size, intervention focus, relevant safety culture dimensions, quality improvement strategies, outcome measures, and main findings.

Author (Year)	Country / Setting	Study Design	Sample Size / Population	Intervention or Focus	Safety Culture Dimension	Quality Improvement Strategy	Outcome Measures	Main Findings
Akcan Yildiz et al., [11] (2024)	Turkey	QI initiative	Children with croup; pediatric residents	Education on croup care with reminders	Evidence-based, safe care practices	Staff training, reminders, audit-feedback	↓ NE use, ↓ X-rays, ↑ dexamethasone, ↓ antibiotics/labs, ↓ LOS, same revisit rate	NE: 80.2%→36.3%; X-rays: 37.4%→17.1%; ↑ dexamethasone; ↓ antibiotics/tests; shorter LOS; revisits unchanged
Park et al., [12] (2023)	USA / Single academic, quaternary-care pediatric ED	Quality Improvement Initiative	818 ED encounters (292 pre, 526 post)	Standardizing bladder filling process for TPUS	Process efficiency, patient safety	Plan-Do-Study-Act cycles; new electronic order set; bladder scan by nurses; IV fluid optimization	Time to TPUS, ED length of stay, repeat TPUS rate	Time to TPUS decreased (138→120 min), ED LOS decreased (372→335 min), repeat TPUS decreased (18%→4%)
Paluck et al., [13] (2023)	Canada	QI / Interventional	Children 6–24 mo	Urine bag UTI screening	Staff engagement, leadership support	PDSA cycles, interprofessional team	Catheterization rate, urine cultures, ED LOS, RVs	Catheterizations ↓ 73.3%→37.7%; urine cultures ↓ 79.8%→40.7%; LOS ↑ 17 min; RVs unchanged
Nucci et al., [14] (2022)	Italy	Quasi-experimental, uncontrolled before-after	885 children with forearm fractures	BST-PSA course to enable PSA in PED	Staff competence and procedural safety	Training-based QI intervention	PSA usage rate, hospitalization rate, adverse events, cost savings	PSA use increased from 37% to 85.3%; hospitalizations decreased from 68.2% to 31.8%; cost savings €370,714; no serious adverse events
Mullan et al., [15] (2023)	USA / Children's Hospital ED & inpatient	Pre-post	≤18 y, MBH pts, 494 alerts	MBH Clinical Debriefing	Teamwork, standards	Debrief after events	2PR rate, ED PR duration, ED PR time, LOS, MMV, themes	ED 2PR ↓67%, ED PR duration ↓112→71 min, ED PR time ↓82%, LOS & MMV ↑, themes: teamwork 23%, standards 14%, inpatient outcomes unchanged
McKinley et al., [16] (2020)	USA, New York City, large urban academic pediatric ED	Simulation study (based on prospective and retrospective data)	Children with cancer and central venous catheters presenting with fever	Time-specific protocol to decrease time to antibiotic delivery	System flow resilience	Discrete event simulation to test QI protocol	WT, LOS	Protocol had no significant impact on patient flow; system showed resilience, with WT increase only at high protocol uptake
Lipshaw et al., [17] (2021)	USA / Large urban pediatric tertiary care center	Prospective QI project	Patients with constipation	Standardized evaluation & management algorithm, EMR integration, educational conferences, discharge instructions	Teamwork, learning culture, standardization	Key driver diagram, statistical process control charts, educational interventions	AXR utilization, length-of-stay, 7-day return visits, inpatient admissions	AXR utilization decreased from 49.6% to 37.1%; length-of-stay, return visits, and admissions unchanged
Katz-Dana et al., [18] (2024)	Canada	Prospective QI study	29 (“POCUS-first” group), 70 (non-POCUS group); children with ileocolic intussusception	Implementation of a “POCUS-first” pathway for intussusception	Streamlined workflow; timely assessment	POCUS-first pathway; physician training, education, PDSA	Physician assessment to reduction time, ED length of stay	“POCUS-first” pathway reduced assessment to reduction time and ED length of stay compared to standard care
Furmick et al., [19] (2022)	USA (Children’s ED)	Uncontrolled before-and-after study	215 children (118 before, 97 after)	Implementation of a point-of-care urinalysis pathway	NR	Pathway-driven point-of-care urine testing to guide treatment and disposition	Time from urinalysis order to discharge order, ED length of stay, rate of delayed treatment	Point-of-care urine testing reduced median time from urinalysis order to discharge by 30 minutes and ED length of stay by 36 minutes; no delayed treatment occurred
Foster et al., [20] (2020)	USA / Pediatric ED & Hospital Medicine	Pre-post QI study	168 encounters (65 baseline, 103 intervention)	CPG for febrile infants	Team collaboration	Education, audit/feedback, electronic orderset	Risk stratification, length of stay	Improved risk stratification; inappropriate admissions ↓14.8%→10.8%; high-risk LOS ↓49.4→38.2 hrs
De Leon et al., [21] (2020)	USA / Pediatric Level I Trauma Center	Historical control, posttest	180 patients (90/90)	Alternate care site for surge	Patient flow, timely care	Activate alternate care site	Census, LOS (admit/discharge), LWBS, hours per visit, patient satisfaction	Reduced wait times, LOS, LWBS; hours per visit and satisfaction unchanged
Carney et al., [22] (2020)	USA	QI / Lean Implementation	70,088	Redesign of ED front-end processes: Flow Nurse/EMT roles, quick registration, direct-bedding, physician-led Intake	Teamwork, Communication, Process Awareness	Lean methodology, Quality Improvement	Door-to-provider time, LWBS rate, % patients seen <30 min, ED length-of-stay, patient satisfaction, 72-hour return rate	Median door-to-provider time ↓ 49% (25 min), LWBS ↓ 56%, % seen <30 min ↑, overall ED LOS ↓, patient satisfaction ↑, no worsening in 72-hour returns
Bram et al., [23] (2021)	USA / Pediatric Emergency Department of a tertiary care children’s hospital	QI initiative	1052 and 2204	Implementation of sterile blood culture collection process with nursing education and individualized feedback	Teamwork, accountability, adherence to safe clinical practices	Process standardization, staff education, performance feedback	Primary: % of contaminated blood cultures; Process: nurse adherence to sterile steps; Balancing: antibiotic administration time and ED stay	Contamination reduced from 6.7% to 2.1%; 98% nurses adhered to ≥75% of sterile steps; no delay in antibiotic administration or ED stay
Baumer-Mouradian et al., [24] (2020)	USA (Pediatric Emergency Department)	QI study using PDSA cycles	33,311 pediatric patients	Implementation of influenza vaccination program including vaccine screening, protocol creation, staff education, pharmacy workflow revision, and weekly feedback	Teamwork, communication, staff engagement, patient safety	PDSA cycles, process mapping, staff education, protocol standardization, feedback loops	% patients screened, # vaccines administered, ED LOS, vaccine waste, financial impact	Screening improved from 0% to 90%; 1,323 vaccines administered; no increase in LOS or financial loss; program was efficient and cost-effective

Key Quality Improvement Interventions and Integration Approaches

The interventions implemented across the studies can be broadly categorised into several key integration approaches. A prominent strategy was the standardisation of clinical processes through the implementation of clinical pathways, algorithms, and order sets. For instance, studies successfully introduced standardised pathways for constipation management [17], urinary tract infection (UTI) diagnosis using point-of-care testing [19], and evaluation of ovarian torsion [12], all of which aimed to reduce unwarranted practice variation and promote evidence-based care.

Another common approach was education and training initiatives designed to enhance staff competence and adherence to best practices. These included structured education for residents on croup management [11], simulation-based training for procedural sedation and analgesia [14], and targeted training for nurses on sterile blood culture collection techniques [23]. Furthermore, several studies leveraged technological integration to support these efforts, such as the incorporation of "POCUS-first" pathways for intussusception into clinical workflow [18] and the development of electronic order sets to standardise bladder filling for pelvic ultrasounds [12].

System-level process redesign was also a significant theme. Studies like those by Carney et al. [22] and De Leon et al. [21] focused on reorganising the front-end operations of the PED and establishing alternate care sites, respectively, to improve patient flow and manage surges. Finally, structured team communication and feedback mechanisms were employed to foster a culture of safety and continuous learning. This was exemplified by the implementation of clinical debriefing processes following behavioural health emergencies to reduce physical restraint use [15] and the use of audit-and-feedback to reinforce sterile procedures and improve vaccination screening rates [23,24]. A summary of the integration approaches, along with the tools and contextual factors involved, is provided in Table 3.

**Table 3:** Summary of key findings across studies This table synthesises key operational aspects from the included studies, including the specific integration approach used, measurement tools employed, and the identified barriers and facilitators to implementation.

Author (Year)	Integration Approach	Measurement Tools Used	Barriers Identified	Facilitators / Enablers
Akcan Yildiz et al., [11] (2024)	Education + reminders for residents to reduce over-testing	NE use, X-rays, dexamethasone, antibiotics, labs, LOS	High rates of unnecessary interventions remain	Education, reminders, protocols, monitoring
Park et al., [12] (2023)	Standardizing bladder filling process using sequential PDSA cycles	Statistical process control charts; monthly mean time to TPUS; ED length of stay; percentage of repeat TPUS	Time-consuming bladder filling leading to delays, poor visualization, prolonged ED LOS	New electronic order set; bladder scan by ED nurses; sequential PDSA cycles; iterative process improvements
Paluck et al., [13] (2023)	Urine bag screening with POC urinalysis using PDSA cycles	Catheterization rate, urine culture rate, ED LOS, return visits	Slight LOS increase, workflow change	Nursing champion, stakeholder engagement, strategic alignment, provider buy-in
Nucci et al., [14] (2022)	Implementation of a BST-PSA course to enable PSA use in Pediatric Emergency Department	Historical control and prospective analysis; Statistical tests: Fisher exact test, Pearson χ² test	NR	Training program; Improved patient flow; Increased PSA use; Reduced hospitalizations; Cost savings; Safety maintained with no serious adverse events
Mullan et al., [15] (2023)	MBH clinical debriefing after behavioral events	2PR rate, ED PR duration, LOS, MMV, qualitative themes	Limited inpatient impact and partial adoption	Team cooperation and adherence to clinical standards
McKinley et al., [16] (2020)	Time-specific antibiotic protocol	Simulation model, ED data	Increased WT for some patients	System resilience, safe testing
Lipshaw et al., [17] (2021)	Standardized algorithm, EMR integration, educational sessions	Statistical process control chart	NR	Multidisciplinary team, QI methods, standardized interventions
Katz-Dana et al., [18] (2024)	Implementation of a “POCUS-first” pathway in Pediatric ED for suspected intussusception	Time from physician initial assessment to radiology-performed US; time from physician initial assessment to reduction; ED LOS	Implicit barriers include reliance on radiology-performed ultrasound, potential training gaps, workflow delays	Pediatric emergency medicine physician training, education, pathway dissemination, continuous improvement via PDSA cycles
Furmick et al., [19] (2022)	Implementation of a point-of-care urine testing pathway to guide UTI management in pediatric ED	Point-of-care urinalysis; comparison with urine microscopy; outcome measures included time from urinalysis order to discharge order, overall ED length of stay, and rate of delayed treatment	No major barriers explicitly reported; potential implicit barrier could be change in workflow from standard urine microscopy	Reduced length of stay and faster treatment decisions; pathway-driven decision-making; prospective evaluation supporting adoption
Foster et al., [20] (2020)	CPG implementation via Model for Improvement; collaboration between pediatric emergency and hospital medicine; interventions: education, audit/feedback, electronic orderset	Statistical process control charts	NR	Collaboration across specialties; structured interventions; quality improvement methodology
De Leon et al., [21] (2020)	Alternate care site to manage patient surges	Census, LOS (admissions/discharges), left without being seen, patient satisfaction, process metrics	Limited generalizability, seasonal activation	Quick setup, low cost, proximity to ED, maintained patient flow and satisfaction
Carney et al., [22] (2020)	Lean QI redesign of ED front-end, new roles, quick registration, direct-bedding, physician-led Intake	Door-to-provider time, LWBS rate, ED LOS, patient satisfaction, 72-hr return	NR	Inter-professional team, Lean methodology, novel Intake system
Bram et al., [23] (2021)	Sterile blood culture collection integrated with nursing education and feedback to improve safety and quality in the pediatric ED.	Audit cards, contamination rate monitoring, antibiotic timing, and ED stay duration.	Inconsistent adherence to sterile steps; reliance on self-reporting.	Ongoing training, feedback, standardized process, and staff engagement.
Baumer-Mouradian et al., [24] (2020)	Influenza vaccination integrated into pediatric ED workflow using PDSA cycles.	Process mapping, vaccination screening rate, vaccines administered, ED LOS, and cost impact.	Workflow gaps, limited staff training, and possible ED flow disruption.	Team collaboration, clear protocol, staff education, revised pharmacy steps, and regular feedback.

Impact on Safety Culture Dimensions and Patient Outcomes

The integrated QI initiatives demonstrated a positive impact on various dimensions of safety culture and led to measurable improvements in patient care outcomes.

Teamwork and communication were explicitly enhanced in several studies. Mullan et al. [15] reported that post-event debriefing improved interdisciplinary cooperation, while Bram et al. [23] and Baumer-Mouradian et al. [24] highlighted that their successes were facilitated by strong team collaboration and engagement.

Evidence-based and standardised practices were significantly promoted, leading to a reduction in unnecessary and potentially harmful interventions. For example, Akcan Yildiz et al. [11] achieved a substantial decrease in the use of nebulised epinephrine and X-rays for croup, while Lipshaw et al. [17] reduced abdominal X-ray utilisation for constipation. Paluck et al. [13] successfully decreased invasive catheterisation for UTI screening, and Foster et al. [20] reduced inappropriate hospitalisations for febrile infants.

Patient safety and procedural efficiency were consistently improved. Multiple studies reported reductions in time to key interventions or diagnoses, such as time to pelvic ultrasound [12], time to antibiotic delivery [16], and time from assessment to reduction for intussusception [18]. This is often translated into a decreased ED LOS [12,19,22]. Importantly, initiatives also directly enhanced procedural safety, with Nucci et al. [14] increasing the use of procedural sedation without serious adverse events, and Bram et al. [23] dramatically reducing blood culture contamination rates.

Patient flow and system efficiency were key outcomes for several system-level interventions. Carney et al. [22] reported a 49% reduction in door-to-provider time and a 56% decrease in patients leaving without being seen following a front-end redesign. The use of an alternate care site during surges also effectively reduced wait times and maintained patient satisfaction [21].

Facilitators, Barriers, and Implementation Strategies

The successful implementation of these QI initiatives was supported by several key facilitators. As detailed in Table 2, iterative improvement methods like PDSA cycles were crucial for testing changes and refining interventions [12,13,19]. Stakeholder engagement and the presence of clinical champions were repeatedly cited as critical enablers, ensuring provider buy-in and sustaining momentum [13,24]. Furthermore, structured education for staff [11,14,18,23] and the use of audit and feedback mechanisms [11,20,23] were vital for embedding new practices into the clinical culture.

Common barriers included resistance to workflow changes [13,18], the challenge of achieving consistent staff adherence to new protocols [15,23], and potential disruptions to established ED workflows [16,24]. For instance, Katz-Dana et al. [18] noted implicit barriers such as a traditional reliance on radiology-performed ultrasounds, while McKinley et al. [16] found that a new antibiotic protocol could inadvertently increase wait times for some patients under high-uptake scenarios, illustrating the complex interplay of interventions within a dynamic system.

Risk of Bias (Quality) Assessment of Included Studies

The methodological quality of the 14 included quality improvement studies [11-24] was assessed using the QI-MQCS. Overall, the studies demonstrated strong reporting in foundational domains: all studies clearly articulated their organisational motivation and intervention rationale [11-24], utilised an appropriate QI study design with a well-described comparator [11-24], and consistently reported on relevant health outcomes and limitations [11-24]. However, several key areas exhibited common reporting weaknesses. Specifically, measurement and reporting of implementation fidelity and adherence were variable, with only five studies [12,13,19,23,24] providing substantial evidence, while others reported this only partially [11,15,17,18,20-22] or not at all [14]. Furthermore, assessments of organisational readiness prior to implementation were often absent [11,14,16,18] or only partially described [12,13,15,17,19-23], and while most studies documented the penetration of their intervention [11-15,17-24], evidence of sustainability beyond the study period was limited, with no study providing strong evidence and the majority reporting none [11,13,15,16,18-20] or only partial evidence [12,14,17,21-24]. Crucially, no study reported on the spread of the intervention to other units or contexts [11-24] (Table 4).

**Table 4:** Quality assessment of included studies using the quality improvement minimum quality criteria set (QI-MQCS) This table presents the methodological quality appraisal of the 14 included studies across the 16 domains of the QI-MQCS tool, indicating whether each criterion was fully met (Yes), partially met (Partial), not met (No), or not applicable (N/A).

QI-MQCS Domain	Akcan Yildiz et al., [11] (2024)	Park et al., [12] (2023)	Paluck et al., [13] (2023)	Nucci et al., [14] (2022)	Mullan et al., [15] (2023)	McKinley et al., [16] (2020)	Lipshaw et al., [17] (2021)	Katz-Dana et al., [18] (2024)	Furmick et al., [19] (2022)	Foster et al., [20] (2020)	De Leon et al., [21] (2020)	Carney et al., [22] (2020)	Bram et al., [23] (2021)	Baumer-Mouradian et al., [24] (2020)
1. Organizational Motivation	Yes	Yes	Yes	Yes	Yes	Yes	Yes	Yes	Yes	Yes	Yes	Yes	Yes	Yes
2. Intervention Rationale	Yes	Yes	Yes	Yes	Yes	Yes	Yes	Yes	Yes	Yes	Yes	Yes	Yes	Yes
3. Intervention Description	Yes	Yes	Yes	Yes	Yes	Partial	Yes	Yes	Yes	Yes	Yes	Yes	Yes	Yes
4. Organisational Characteristics	Partial	Yes	Partial	Partial	Yes	Partial	Yes	Partial	Yes	Yes	Yes	Yes	Yes	Yes
5. Implementation Strategy	Yes	Yes	Yes	Yes	Yes	Partial	Yes	Yes	Yes	Yes	Yes	Yes	Yes	Yes
6. Study Design	Yes	Yes	Yes	Yes	Yes	Yes	Yes	Yes	Yes	Yes	Yes	Yes	Yes	Yes
7. Comparator Description	Yes	Yes	Yes	Yes	Yes	Yes	Yes	Yes	Yes	Yes	Yes	Yes	Yes	Yes
8. Data Source	Yes	Yes	Yes	Yes	Yes	Yes	Yes	Yes	Yes	Yes	Yes	Yes	Yes	Yes
9. Timing	Yes	Yes	Yes	Yes	Yes	Yes	Yes	Yes	Yes	Yes	Yes	Yes	Yes	Yes
10. Adherence/Fidelity	Partial	Yes	Yes	No	Partial	N/A	Partial	Partial	Yes	Partial	Partial	Partial	Yes	Yes
11. Health Outcomes	Yes	Yes	Yes	Yes	Yes	Yes	Yes	Yes	Yes	Yes	Yes	Yes	Yes	Yes
12. Organizational Readiness	No	Partial	Yes	No	Partial	No	Partial	No	Partial	Partial	Partial	Partial	Partial	Yes
13. Penetration/Reach	Yes	Yes	Yes	Yes	Yes	Partial	Yes	Yes	Yes	Yes	Yes	Yes	Yes	Yes
14. Sustainability	No	Partial	No	Partial	No	No	Partial	No	No	No	Partial	Partial	Partial	Partial
15. Spread	No	No	No	No	No	No	No	No	No	No	No	No	No	No
16. Limitations	Yes	Yes	Yes	Yes	Yes	Yes	Yes	Yes	Yes	Yes	Yes	Yes	Yes	Yes

Discussion

This systematic review synthesised evidence from 14 QI studies to elucidate the strategies and outcomes associated with integrating safety culture principles into QI initiatives within PEDs. The findings collectively demonstrate that a deliberate focus on core safety culture components, such as teamwork, standardised processes, staff engagement, and continuous learning, is not merely complementary but foundational to the success of QI efforts aimed at enhancing patient care. The included studies, despite their heterogeneity in clinical focus, consistently reveal that successful interventions are those that move beyond a purely technical fix and embed change within the social and cultural fabric of the ED. For instance, the most impactful initiatives, such as the clinical debriefing process implemented by Mullan et al. [15] to reduce physical restraints, explicitly targeted psychological safety and interdisciplinary communication, thereby addressing the underlying cultural norms that permit restrictive practices. Similarly, the successes of Bram et al. [23] in reducing blood culture contamination and Baumer-Mouradian et al. [24] in launching an ED vaccination programme were critically dependent on fostering accountability and collective ownership among nursing staff, underscoring that procedural adherence is a cultural artefact as much as a technical skill.

The efficacy of standardised pathways and protocols, as seen in the management of constipation [17], febrile infants [20], and suspected intussusception [18], underscores a central theme: reducing unwarranted clinical variation is a potent mechanism for enhancing both safety and efficiency. These findings align strongly with the broader literature on clinical standardisation. For example, a seminal study by Alessandrini et al. [25] on implementing a paediatric asthma clinical pathway also demonstrated significant reductions in ED length of stay and improved adherence to evidence-based therapies, mirroring the outcomes achieved by Foster et al. [20] and Lipshaw et al. [17]. Our review extends this understanding by highlighting that the integration of these pathways, through EMR order sets, staff education, and audit-and-feedback, is what activates their potential. This resonates with the work of Grol & Grimshaw [26], who long asserted that the effectiveness of guidelines is determined by the strategies used to implement them. The "POCUS-first" pathway by Katz-Dana et al. [18] is a prime example, where streamlining workflow and providing physician training were as crucial as the pathway itself, leading to significant reductions in time to diagnosis and treatment.

Furthermore, the role of iterative, data-driven QI methodologies, particularly the PDSA cycle, emerged as a critical facilitator across multiple studies [12,13,19,24]. This approach allows for rapid-cycle testing and adaptation, which is essential in the dynamic and high-stakes environment of the PED. The work of Park et al. [12] in standardising the bladder-filling process for pelvic ultrasounds exemplifies this; through sequential PDSA cycles, they were able to refine their intervention, leading to sustained reductions in time to ultrasound and ED length of stay. This aligns with the Model for Improvement, popularised by Langley et al. [27], which has been widely adopted in healthcare to facilitate incremental, sustainable change. Our findings corroborate the value of this model, as used by Foster et al. [20], and suggest that its flexibility is particularly well-suited to cultivating a safety culture, as it empowers frontline staff to identify problems and test solutions, thereby fostering a sense of ownership and a culture of continuous learning.

The impact on specific safety culture dimensions was palpable across the reviewed studies. Teamwork and communication were repeatedly cited as both a target and a facilitator of success. The reduction in physical restraints through debriefing [15] and the improvement in sterile technique through nursing education and feedback [23] are direct reflections of enhanced team coordination and psychological safety. These findings are consistent with earlier research linking safety culture to patient outcomes. A study by Huang et al. [28] in a general ED setting found that higher scores on safety culture surveys, particularly in the domains of teamwork and communication openness, were associated with lower rates of reported medication errors. Our review provides concrete, QI-driven examples that affirm this relationship in paediatrics, demonstrating that cultural elements can be actively cultivated through structured interventions. Moreover, the focus on staff engagement and leadership support in studies like Paluck et al. [13] and Baumer-Mouradian et al. [24] reinforces the findings of Singer et al. [29], who identified leadership commitment and staff involvement as critical predictors of a positive safety culture in hospitals.

However, the QI-MQCS assessment reveals a significant gap between achieving short-term, local success and demonstrating long-term, systemic transformation. The near-universal absence of reporting on sustainability and spread is a critical limitation of the current evidence base. While studies like those of Carney et al. [22] and Nucci et al. [14] showed impressive results in patient flow and cost savings, respectively, it remains unknown whether these improvements were maintained after the formal study period concluded or if they could be replicated in other settings. This is a common challenge in QI literature. A systematic review by Endalamaw et al. [30] on the sustainability of QI programmes in healthcare found that few studies report long-term outcomes, and even fewer describe the factors that promote sustainment. Our findings are congruent with this, highlighting a pervasive "projectification" of QI, where initiatives are treated as time-limited endeavours rather than permanent shifts in practice and culture. The failure to report on spread limits the generalisability and potential impact of these successful interventions. The work of Carney et al. [22] on front-end redesign, for example, represents a potentially transformative model for EDs globally, yet without documented efforts at dissemination and adoption in other contexts, its broader utility remains theoretical.

Limitations

This systematic review has several limitations. First, the included studies themselves possess methodological constraints, as identified by the QI-MQCS assessment. The prevalent use of pre-post designs without concurrent control groups limits the strength of causal inference, as secular trends or other concurrent changes in the PED could account for some of the observed effects. The general lack of long-term follow-up data on sustainability, as previously discussed, means the enduring impact of these interventions remains uncertain. Second, the search was limited to published, peer-reviewed literature, which may introduce publication bias, as successful QI initiatives are more likely to be written up and published than failed or inconclusive ones. Third, the definition and measurement of "safety culture" were not uniform across studies; while some explicitly measured dimensions like teamwork, others inferred cultural impact from process outcomes. This heterogeneity makes direct comparison and synthesis challenging. Finally, the review focused specifically on the PED context, and while the principles of integrating safety culture and QI are likely generalisable, the specific strategies and their effectiveness may differ in other clinical settings.

## Conclusions

The integration of safety culture principles is a powerful catalyst for successful quality improvement in the paediatric emergency department. The synthesis of 14 studies demonstrates that interventions which standardise care, engage and educate frontline staff, leverage iterative improvement methods, and foster teamwork and communication are consistently associated with enhanced patient safety, improved clinical outcomes, and greater operational efficiency. These findings underscore that QI is not merely a technical or procedural exercise but a deeply socio-cultural one. The most meaningful and sustained improvements arise from initiatives that thoughtfully address the human elements of teamwork, psychological safety, and shared accountability. Moving forward, the field must advance beyond demonstrating initial success. Future QI initiatives and their publications must prioritise and rigorously evaluate the long-term sustainability of improvements and develop robust strategies for spreading effective interventions beyond single institutions. By doing so, the PED community can transform isolated successes into an enduring culture of safety and excellence that benefits all children seeking emergency care.

## Appendices

**Table 5:** Detailed search strategy for each databases

Database	Search Strategy
PubMed	("Safety Culture"[Mesh] OR "safety culture"[tiab] OR "safety climate"[tiab] OR "patient safety"[tiab] OR "safety attitude"[tiab]) AND ("Quality Improvement"[Mesh] OR "quality improvement"[tiab] OR "quality initiative"[tiab] OR "QI"[tiab] OR "process improvement"[tiab] OR "PDSA"[tiab] OR "Plan Do Study Act"[tiab]) AND ("Emergency Service, Hospital"[Mesh] OR "pediatric emergency"[tiab] OR "paediatric emergency"[tiab] OR "PED"[tiab] OR "ED"[tiab] OR "emergency department"[tiab]) Filters: Publication date from 2020/01/01 to 2024/12/31; English
Scopus	( TITLE-ABS-KEY ( "safety culture" OR "safety climate" OR "patient safety" OR "safety attitude" ) ) AND ( TITLE-ABS-KEY ( "quality improvement" OR "quality initiative" OR "qi" OR "process improvement" OR "pdsa" OR "plan do study act" ) ) AND ( TITLE-ABS-KEY ( "pediatric emergency" OR "paediatric emergency" OR "ped" OR "emergency department" OR "ed" ) ) Filters: PUBYEAR > 2019 AND PUBYEAR < 2025, English
Web of Science	TS=( ("safety culture" OR "safety climate" OR "patient safety" OR "safety attitude") ) AND TS=( ("quality improvement" OR "quality initiative" OR "QI" OR "process improvement" OR "PDSA" OR "Plan Do Study Act") ) AND TS=( ("pediatric emergency" OR "paediatric emergency" OR "PED" OR "emergency department" OR "ED") ) Filters: Publication Years: 2020-2024, English
Embase	('safety culture'/exp OR 'safety culture':ti,ab,kw OR 'safety climate':ti,ab,kw OR 'patient safety':ti,ab,kw OR 'safety attitude':ti,ab,kw) AND ('quality improvement'/exp OR 'quality improvement':ti,ab,kw OR 'quality initiative':ti,ab,kw OR 'qi':ti,ab,kw OR 'process improvement':ti,ab,kw OR 'pdsa':ti,ab,kw OR 'plan do study act':ti,ab,kw) AND ('pediatric emergency'/exp OR 'pediatric emergency':ti,ab,kw OR 'paediatric emergency':ti,ab,kw OR 'ped':ti,ab,kw OR 'emergency department':ti,ab,kw OR 'ed':ti,ab,kw) Filters: Publication date from 2020-01-01 to 2024-12-31, English
